# Functional polymorphisms in the promoter region of MMP-2 and MMP-9 and susceptibility to obstructive sleep apnea

**DOI:** 10.1038/srep08966

**Published:** 2015-03-10

**Authors:** Chao Cao, Bin Wu, Yanping Wu, Yiming Yu, Hongying Ma, Shifang Sun, Qiaoli Zhang, Qunli Ding, Li Chen, Zaichun Deng

**Affiliations:** 1 Department of Respiratory Medicine, Affiliated Hospital of School of Medicine, Ningbo University, Ningbo 315020, China; 2 Department of Respiratory and Critical Care Medicine, Second Affiliated Hospital, Zhejiang University School of Medicine, Hangzhou 310009, China; 3 Department of Respiratory and Critical Care Medicine, Affiliated Hospital, Institute of Respiratory Diseases, Guangdong Medicine College, Zhanjiang 524000, China; 4 Department of Medical Oncology, First Affiliated Hospital of Nanchang University, Nanchang 330006, China

## Abstract

Genetic susceptibility to obstructive sleep apnea (OSA) has been a research focus in the scientific community in the past few years. In this study, we recruited 375 subjects to investigate whether functional polymorphisms in the promoter region of matrix metalloproteinase (MMP)-2 (-1306C/T) and MMP-9 (-1562C/T) increased susceptibility to OSA. Our study showed no significant association between MMP-2 -1306C/T polymorphism and risk of OSA (T *vs.* C: OR = 1.01, 95% CI = 0.67–1.52; *P* = 0.97). Compared with the MMP-9 -1562C allele, the -1562T allele was associated with increased risk of OSA (T *vs.* C: OR = 1.56, 95% CI = 1.02–2.39; *P* = 0.04). However, neither MMP-2 -1306C/T nor MMP-9 -1562C/T polymorphism was found to be associated with severity of the disease. Our study suggested that the MMP-2 -1306C/T polymorphism was not associated with OSA susceptibility, whereas the MMP-9 -1562T allele was associated with increased risk of OSA.

Obstructive sleep apnea (OSA) is a prevalent sleep disorder characterized by repeated events of partial or complete upper airway obstruction during sleep, which results in intermittent hypoxemia, sleep fragmentation, and daytime sleepiness^1^. OSA has also been associated with cardiovascular diseases, such as hypertension, coronary heart disease, heart failure, stroke, cardiac arrhythmias, and sudden cardiac death^2^. OSA is a common disease with high morbidity in both adults and children, and the underlying mechanism of the disease remains unknown. In the last few years, a growing body of evidence has suggested that matrix metalloproteinases (MMPs) are involved in the pathogenesis of OSA^3^,^4^,^5^,^6^,^7^,^8^,^9^,^10^,^11^,^12^. A common polymorphism of MMP-2 gene is (-1306C/T) [rs243865], which is found to be associated with a strikingly lower activity with the T allele^13^. At the same time, there is evidence that -1562C/T polymorphism in the promoter region of MMP-9 [rs3918242] is associated with its mRNA transcription and protein production^14^. However, the relationship between MMP-2 and MMP-9 polymorphisms and risk of OSA has not been investigated worldwide. Based on the pathologic significance of MMPs in OSA and the potential biological effects of MMP-2 (-1306C/T) and MMP-9 (-1562C/T) polymorphisms on their protein expression, we hypothesized that functional polymorphisms in the promoter region of MMP-2 and MMP-9 would be associated with differential risk of OSA. Therefore, we conducted a case-control study to investigate whether polymorphisms of MMP-2 (-1306C/T) and MMP-9 (-1562C/T) increased susceptibility to OSA in a Chinese population.

## Results

### Genotype frequencies and risk of OSA

The MMP-2 -1306C/T and MMP-9 -1562C/T genotypes observed in controls were consistent with Hardy–Weinberg equilibrium (HWE) (*P* > 0.05, chi-squared goodness of fit). Allele frequencies of MMP-2 -1306C and MMP-9 -1562C polymorphisms in the controls were 0.85 and 0.89, respectively, which were in close agreement with frequencies previously observed in healthy Chinese individuals (0.83 and 0.90, respectively)^15^. The genotype distributions and allele frequencies of MMP-2 -1306C/T and MMP-9 -1562C/T polymorphisms among OSA patients and controls were shown in Table 1. There was no significant association between the MMP-2 -1306C/T polymorphism and risk of OSA (T *vs.* C: OR = 1.01, 95% CI = 0.67–1.52; *P* = 0.97), whereas the MMP-9 -1562T genotype was associated with an increased risk of OSA (T *vs.* C: OR = 1.56, 95% CI = 1.02–2.39; *P* = 0.04).

### Genotypes and severity of OSA

The effect of MMP-2 -1306C/T and MMP-9 -1562C/T genotypes on OSA severity was further assessed. There were 82 patients with mild-to-moderate OSA and 68 with severe OSA. The genotype distributions were similar between the non-severe group and severe group. As shown in Table 2, neither of the two polymorphic genotypes showed a significant difference between mild-to-moderate OSA patients and patients with severe OSA.

## Discussion

Genetic susceptibility to OSA has been a research focus in the scientific community in the past few years. Numerous molecular epidemiological studies have identified several genetic variants as biomarkers for genetic susceptibility to OSA^15^,^16^,^17^,^18^,^19^,^20^,^21^. Moreover, in our previous study, we even observed a functional EGF+61A/G polymorphism was associated with the severity of OSA^22^. In the present study, we conducted a case-control study with 150 OSA patients and 225 controls to investigate the association between functional polymorphisms in the promoter region of MMP-2 and MMP-9 and risk of OSA. To the best of our knowledge, this is the first study to investigate these two polymorphisms and susceptibility to OSA.

The C/T transition at -1306 is a common polymorphism observed in MMP-2 and is known to disrupt a Sp1-type promoter site (CCACC box) leading to lower promoter activity associated with the T allele^23^. However, we did not find any significant difference in genotypic and allelic distribution between the control group and the OSA group. In addition, our study showed little association between MMP-2 -1306C/T polymorphism and risk of OSA in any of the genetic models tested (T *vs.* C: OR = 1.01, 95% CI = 0.67–1.52). In stratified analysis by severity of disease, similar results were obtained (TT *vs.* CC: OR = 0.84, 95% CI = 0.05–13.86).

Interestingly, subjects with the MMP-9 -1562T allele were associated with an increased risk of OSA when compared with -1562C carriers (OR = 1.56, 95% CI = 1.02–2.39). Significant associations between the MMP-9 -1562C/T polymorphism and risk of OSA were also observed under heterozygote comparison (CT *vs.* CC: OR = 1.86, 95% CI = 1.15–3.01) and dominant genetic model (CT/TT *vs.* CC: OR = 1.78, 95% CI = 1.11–2.85). One possible explanation for these results is that DNA sequence variations in the MMP-9 gene may alter its protein production and/or activity. The investigators observed that T allele-associated promoter activity was higher than the C allele-associated promoter activity by transient transfection and DNA-protein interaction assays^14^. Moreover, published evidence has demonstrated that a genetic variant in the region of the MMP-9 -1562C/T polymorphism is associated with serum levels of MMP-9^24^,^25^,^26^. The MMP-9 -1562 C/T polymorphism associated with a predisposition to increased serum MMP-9 levels. Thus, it is biologically plausible that the functional polymorphism in this gene increases circulating MMP-9 expression, thereby causing individual differences in the development of OSA.

Some limitations of this study should be acknowledged. Firstly, in this study, we only investigated two functional polymorphisms in the promoter region of MMP-2 and MMP-9 with the risk of OSA. The association between other polymorphisms in MMP-2 and MMP-9 and their relationships to OSA susceptibility requires further study. Secondly, the sample size in the subgroup analysis of the severity of OSA was not large enough. Therefore, the power was very limited and it could just be underpowered to detect an association. Additional studies with larger sample sizes are warranted to validate our findings.

In summary, our study showed that the MMP-2 -1306C/T polymorphism was not associated with OSA susceptibility, whereas the MMP-9 -1562T allele was associated with an increased risk of OSA. These results need to be validated by other independent studies. Future studies should be conducted in diverse ethnic populations.

## Methods

### Participants

Consecutive patients with suspected sleep apnea that were undergoing polysomnography (PSG) test in the Respiratory Department of Affiliated Hospital of Ningbo University were invited to participate in this study. Approval for this study was obtained from the Institutional Review Board for Human Studies of Affiliated Hospital, School of Medicine, Ningbo University (Ningbo, China), and written informed consent was obtained from all participating subjects. The experiments were performed in accordance with American Academy of Sleep Medicine Guidelines^1^. Each participant was assessed by a detailed clinical interview and physical examination. Severity of disease was assessed based on the apnea hypopnea index (AHI), defined as the mean number of apneas and hypopneas per hour during sleep. OSA patients were categorized into 2 groups based on AHI: mild-to-moderate OSA (5 ≤ AHI < 30) and severe OSA (AHI ≥ 30). A total of 150 patients (F36: M114; age: 48.7 ± 11.5 years) with OSA and 225 healthy controls matched for age, sex, and ethnicity were included in this study. Base on AHI values, there were 82 patients with mild-to-moderate OSA (54.7%) and 68 with severe OSA (45.3%).

### Polysomnography (PSG)

All the subjects underwent an overnight laboratory-based PSG, which was analyzed according to recommendations published by the American Academy of Sleep Medicine^1^. Measurement channels included nasal pressure airflow, oxygen saturation (SpO_2_), heart rate, electroencephalogram, electrocardiogram, electrooculogram, electromyo-gram (chin and leg), snoring, and chest and abdominal movements. Participants went to bed before 23:00, and the recording was terminated after 6:00. The records were reviewed manually by trained sleep technologists for sleep stage, leg movements, arousals, apneas, and hypopneas. Records showing ≧ 4 hours of good quality respiratory signals were considered acceptable.

### DNA isolation and genotyping assays

Five milliliters (5 ml) of venous blood from each case and control subjects were collected in EDTA-containing tubes. Genomic DNA was extracted from whole blood by using a commercially available DNA isolation kit (Tiangen, Beijing, China) according to the manufacturer's protocol. Then, the purity of DNA was measured by means of absorption spectrometry, and the samples with absorbance rations from 1.8 to 2.0 at the length of A260/A280. The MMP-2 (-1306C/T) and MMP-9 (-1562C/T) polymorphisms were determined by polymerase chain reaction - restricted fragment length polymorphism (PCR-RFLP) assays as previously described^27^,^28^. Briefly, PCR reactions were performed in a 20 μl reaction volume and cycle conditions consisted of an initial denaturation step at 94°C for 5 min, followed by 30 cycles of 30 s at 94°C, 30 s at 62°C, 30 s at 72°C, and then a final elongation step at 72°C for 5 min. The restriction enzymes for MMP-2 -1306C/T and MMP-9 -1562C/T genotypes were *Bst* XI and *Nla* III, respectively. PCR products were electrophoresed on a 3% agarose gel and visualized under UV light after staining with ethidium bromide.

### Statistical analysis

The numbers observed for each genotype were compared with those expected for Hardy–Weinberg equilibrium using the χ^2^ test. The results were presented as the means ± standard deviation (SD) for all variables that were normally distributed and as median (interquartile range) when not normally distributed. If the data distribution was normal, comparison between different groups was done using Student's t-test; otherwise, the nonparametric Mann-Whitney U-test was applied. The OSA risk associated with MMP-2 -1306C/T and MMP-9 -1562C/T genotypes was estimated by computing the odds ratios (ORs) and their 95% confidence intervals (CIs) by logistic regression analysis. Statistical analyses were conducted using SPSS (version 13.0, Chicago, III., United States) and GraphPad Prism 5.0 (GraphPad Software, San Diego, CA). A two-tailed *P* value < 0.05 was considered statistically significant.

## Author Contributions

C.C., Z.D. and L.C. designed the experiments. C.C., B.W., Y.W., Y.Y., H.M., S.S., Q.Z., Q.D., Z.D. and L.C. carried out the experiments and calculations. C.C., B.W., Y.W., Z.D. and L.C. wrote and edited the paper.

## Figures and Tables

**Table 1:** MMP-2 -1306C/T and MMP-9 -1562C/T genotype and allele frequency in OSA patients and controls

Polymorphisms	Patients (%)	Controls (%)	OR (95% CI)	*P* value
MMP-2 -1306C/T				
C/C	107 (71.3)	164 (72.9)	1.00 (Reference)	
C/T	41 (27.4)	55 (24.4)	0.89 [0.56, 1.41]	0.61
T/T	2 (1.3)	6 (2.7)	0.51 [0.10, 2.58]	0.42
C/T and T/T	43 (28.7)	61 (27.1)	1.08 [0.68, 1.71]	0.74
Allele				
C	255 (85)	383 (85.1)	1.00 (Reference)	
T	45 (15)	67 (14.9)	1.01 [0.67, 1.52]	0.97
MMP-9 -1562C/T				
C/C	103 (68.7)	179 (79.6)	1.00 (Reference)	
C/T	46 (30.6)	43 (19.1)	1.86 [1.15, 3.01]	0.01
T/T	1 (0.7)	3 (1.3)	0.58 [0.06, 5.64]	0.64
C/T and T/T	47 (31.3)	46 (20.4)	1.78 [1.11, 2.85]	0.02
Allele				
C	252 (84)	401 (89.1)	1.00 (Reference)	
T	48 (16)	49 (10.9)	1.56 [1.02, 2.39]	0.04

MMP: matrix metalloproteinase; OSA: obstructive sleep apnea; OR = odds ratio; CI = confidence interval.

**Table 2:** MMP-2 -1306C/T and MMP-9 -1562C/T genotype with severity of OSA

Polymorphisms	Non-severe (%)	Severe (%)	OR (95% CI)	*P* value
MMP-2 -1306C/T				
C/C	58 (70.7)	49 (72.0)	1.00 (Reference)	
C/T	23 (28.1)	18 (26.5)	1.08 [0.52, 2.23]	0.84
T/T	1 (1.2)	1 (1.5)	0.84 [0.05, 13.86]	0.91
MMP-9 -1562C/T				
C/C	57 (69.5)	46 (67.6)	1.00 (Reference)	
C/T	24 (29.3)	22 (32.4)	0.88 [0.44, 1.77]	0.72
T/T	1 (1.2)	0 (0)	NA	NA

MMP: matrix metalloproteinase; OSA: obstructive sleep apnea; OR = odds ratio; CI = confidence interval; NA: not applicable.
